# Cu_2−*x*_Se Modification onto Monoclinic BiVO_4_ for Enhanced Photocatalytic Activity Under Visible Light

**DOI:** 10.1186/s11671-019-2929-7

**Published:** 2019-03-14

**Authors:** Zirui Liu, Xiaoyong Liu, Xinxin Gu, Renqing Guo, Wenwu Zhong

**Affiliations:** 1 School of Automotive Engineering, Chongqing University, Chongqing, 400044 China; 2 School of Material Science and Engineering, Chongqing University, Chongqing, 400044 China; 3 Department of Materials, Taizhou University, Taizhou, 318000 China

**Keywords:** Photocatalysis, Hydrothermal, Bismuth-based semiconductor

## Abstract

The rapid recombination of electron-hole pairs in BiVO_4_ has limited its performance as a photocatalysis. In this paper, BiVO_4_ is combined with Cu_2−*x*_Se semiconductor to slow down the recombination process, and thus improve its photocatalytic activity. This is enabled by careful band structure design. The work function of Cu_2−*x*_Se is larger than that of BiVO_4_. Therefore, electrons flow to Cu_2−*x*_Se from BiVO_4_ after the composition. Accordingly, an inner field could be built, which facilitates the separation of electrons and holes. The experimental result shows that the photocatalytic efficiency of the 3 wt% Cu_2−*x*_Se/BiVO_4_ composite is 15.8 times than that of pure BiVO_4_.

## Introduction

With the developing of modern industry, environmental pollution has become more and more severe. Utilizing solar energy, photocatalytic decomposition of organic matter is an environmentally friendly and efficient technology to solve pollution [1–6]. The Bi-based semiconductor photocatalytic material has a suitable band gap, which enables it to absorb visible light sufficiently and possess superior photocatalytic performance [7–10]. Among them, monoclinic BiVO_4_ has a narrow band gap of 2.4 eV and good photocatalytic activity, which has been nominated as an efficient material for decomposing organic pollutions [11–15]. The rapid electron-hole recombination rate, however, leads to a low photocatalytic activity for pure BiVO_4_ [16–18]. An effective approach to slow down the recombination of electrons and holes is to combine two different semiconductor materials, given the band structures of the two combined materials match a specific condition.

As a p-type semiconductor, Cu_2−*x*_ Se has an indirect bandgap of 1.4 eV, which is beneficial to absorb visible light [19–21]. When BiVO_4_ semiconductor is compounded with Cu_2−*x*_Se, redistribution of charges is caused. The work function of Cu_2−*x*_Se is larger than that of BiVO_4_, and the Fermi energy is lower than that of BiVO_4_ [22, 23]. Therefore, electrons flow to Cu_2−*x*_Se from BiVO_4_ while holes flow the other way around. Accordingly, an inner field could be built pointing from BiVO_4_ to Cu_2−*x*_Se, which facilitates the separation of electrons and holes. When under illumination, the photo-generated electrons in BiVO_4_ and photo-generated holes in Cu_2−*x*_Se will recombine preferentially, due to the band bending and inner field, leaving useful holes in BiVO_4_. The useful holes possess higher energy level, which can benefit the generation of •OH species. These •OH species can break down long chains of organic matter into small molecules. Hence, the Cu_2−*x*_Se/BiVO_4_ composites are expected to have high visible light photocatalytic activity.

In this work, we have fabricated Cu_2−*x*_ Se/BiVO_4_ composites and made use of it for the degradation of RhB under visible light irradiation (> 420 nm) for the first time. After compounding with Cu_2−*x*_ Se, the photocatalytic activity becomes much higher than pure BiVO_4_. Specifically, the photocatalytic efficiency of 3 wt% Cu_2−*x*_Se/BiVO_4_ composite is 15.8 times that of pure BiVO_4_. Furthermore, after adding low concentration H_2_O_2_ into the organic solution, RhB completely degraded within 50 min. This work provides evidence that Cu_2−*x*_Se is an effective co-catalysis for the development of new composite semiconductor photocatalysts.

## Methods

### Preparation of Cu_2−*x*_Se/BiVO_4_ Composites

**Fig. 1:**
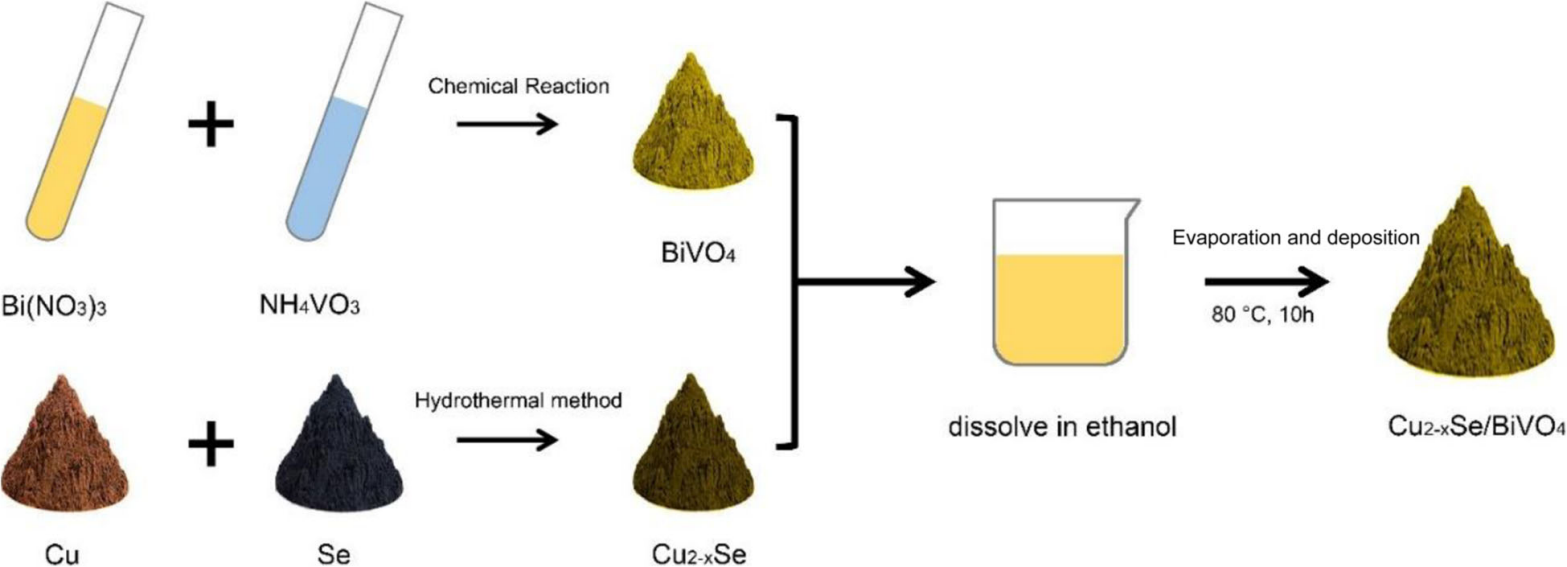
The schematic diagram of formation for Cu_2−*x*_Se/BiVO_4_ composite

BiVO_4_ was synthesized through a chemical precipitation method [24, 25]. The preparation method of Cu_2−*x*_Se can be found in our previously reported paper [26]. Then Cu_2−*x*_Se/BiVO_4_ composites were fabricated by a co-precipitation approach. The schematic illustration of the preparation progress is shown in Fig. 1. Firstly, the pre-prepared Cu_2−*x*_Se and BiVO_4_ powders were dispersed in ethanol with constant stirring for 4 h under 60 °C. Secondly, the suspension of the mixture was continuously stirred at 80 °C to remove the ethanol solvent. Finally, the obtained powdery sample was heated at 160 °C for 6 h under a flowing nitrogen atmosphere to form the Cu_2−*x*_Se/BiVO_4_ composite.

### Characterization

XRD (X-ray diffraction) measurement of the as-prepared samples was performed by a PANalytical X’pert Pro diffractometer with Cu Kα radiation. The morphology of the sample was obtained by an SEM (scanning electron microscope) Hitachi S-4800. XPS (X-ray photoelectron spectroscopy) of the samples was characterized on a Pekin Elmer PHI-5300 instrument. The photoluminescence emission spectra of the samples were committed using a Cary Eclipse fluorescence spectrophotometer.

### Photocatalytic Reaction

The photocatalytic performance was characterized by an XPA photochemical reactor. Additionally, a Xe lamp with a power of 500 W and a cut-off wavelength of 420 nm is utilized to simulate natural light, while a solution of test dye RhB is used to mimic organic solutions. During the degradation process, 60 mg Cu_2−*x*_Se composite powder was placed in a 60-mL RhB solution. The suspension was stirred in a dark environment for 2 h before light irradiation to realize an adsorption-desorption balance. Then, light illumination is added with stirring remaining and about 6 mL of the suspension was taken out at intervals of 10 min. Subsequently, the suspension was centrifuged twice. The absorbance spectrum of the solution was characterized on a Shimadzu UV-2450 spectrometer.

### Photoelectrochemical Measurements

The photocurrent is measured by a CHI 660E electrochemical workstation. To make the illumination consistent with that in the degradation process, the light source is still selected as a Xe lamp with a power of 500 W and a cut-off wavelength of 420 nm. The photoelectrochemical measurement is detailed as follows. First, 10 mg of the photocatalyst and 20 μL of Nafion solution were ultrasonically dispersed in 2 mL of ethyl alcohol. Then, 40 μL of the above solution was deposited on an ITO conductive glass with 0.196 cm^2^, which was sequentially heated at 200 °C for 1 h to obtain the working electrode. Besides, Pt foil is chosen as the counter electrode. A saturated solution of mercury and mercurous chloride in an aqueous solution of potassium chloride as the reference electrode, and 0 .5M Na_2_SO_4_ solution is used for the electrolyte.

## Results and Discussion

**Fig. 2:**
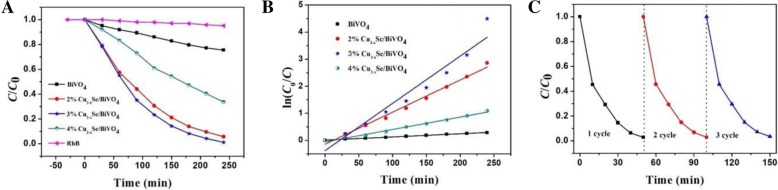
**a** Photocatalytic degradation of RhB over Cu_2−*x*_Se/BiVO_4_. **b** Photocatalytic degradation rate constant of RhB for Cu_2−*x*_Se/BiVO_4_. **c** Recycle runs of photocatalytic degradation of RhB over 3 wt% Cu_2−*x*_Se/BiVO_4_ composite with H_2_O_2_ under visible light irradiation

We used photodegradation of RhB to examine the photocatalytic properties of the samples. Figure 2a shows the photocatalytic degradation of RhB over Cu_2−*x*_Se/BiVO_4_. When BiVO_4_ is combined with Cu_2−*x*_Se, its photocatalytic performance is significantly improved. The optimum composite ratio is 3%, and the photocatalytic efficiency at this ratio reaches the maximum. Figure 2b shows the degradation rate of the Cu_2−*x*_Se/BiVO_4_ composites, corresponding to the concentration of Cu_2−*x*_Se with 0, 2, 3, and 4 wt%, respectively. In Fig. 2b, the slope value of degradation lines is 0.0011, 0.0118, 0.0174, and 0.0045 min^−1^, respectively. Therefore, the photocatalytic efficiency of the 3 wt% Cu_2−*x*_Se/BiVO_4_ composite is 15.8 times than that of pure BiVO_4_. Figure 2c shows the recycle runs of photocatalytic degradation of RhB over 3 wt% Cu_2−*x*_Se/BiVO_4_ composite with added H_2_O_2_ under visible light irradiation. When a small amount of H_2_O_2_ is added (103 μL/100 mL), the 3 wt% Cu_2−*x*_Se/BiVO_4_ composites can degrade RhB completely in 50 min under visible light excitation. It can also be seen from Fig. 2c that the degradation efficiency is not attenuated after 3 cycles.

**Fig. 3:**
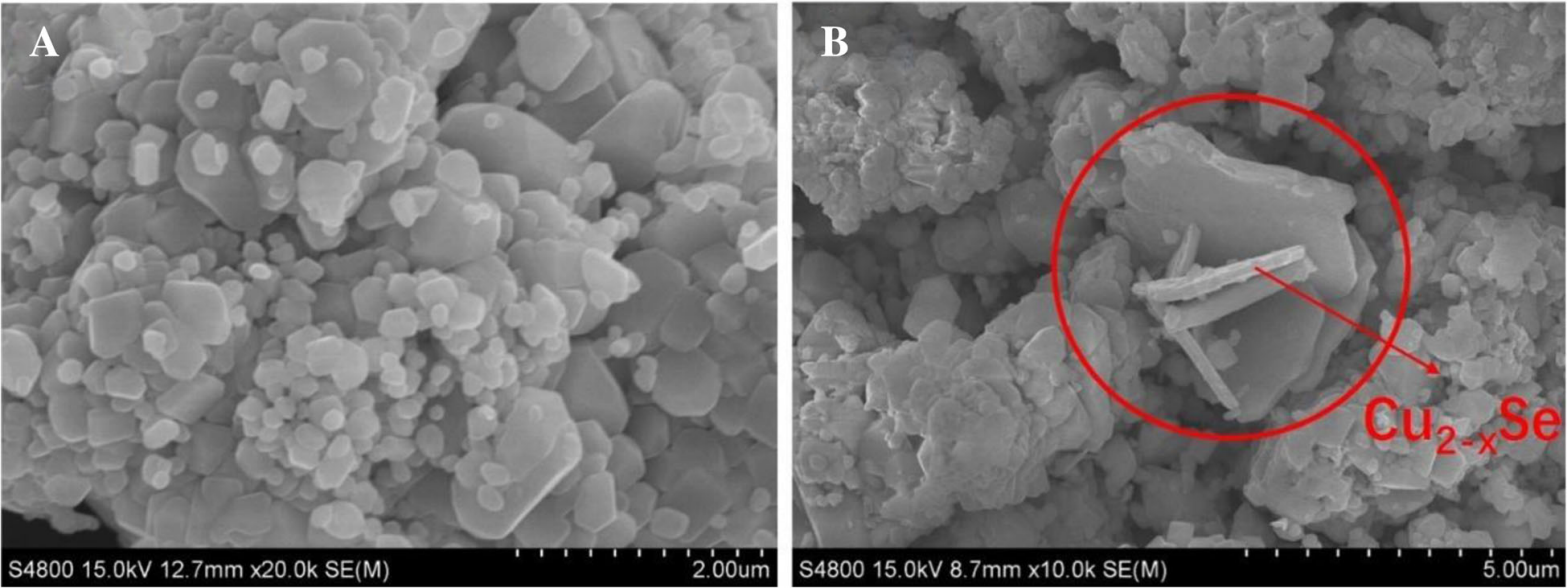
The SEM photograph of BiVO_4_ (**a**) and Cu_2−*x*_Se/BiVO_4_ (**b**)

In order to analyze the microscopic morphology and grain size of the samples, the samples were characterized by SEM. As shown in Fig. 3a, BiVO_4_ is a hexagonal bulk with a particle size of 0.2–1 μm. In Fig. 3b, the area circled by the red solid line exhibits a Cu_2−*x*_Se sheet with a thickness of 300 nm and a length of 4 μm. After compounding, the Cu_2−*x*_Se sheets are randomly distributed on the surface of BiVO_4_ bulk. The XPS results also reveal the presence of Cu_2−*x*_Se (shown below).

**Fig. 4:**
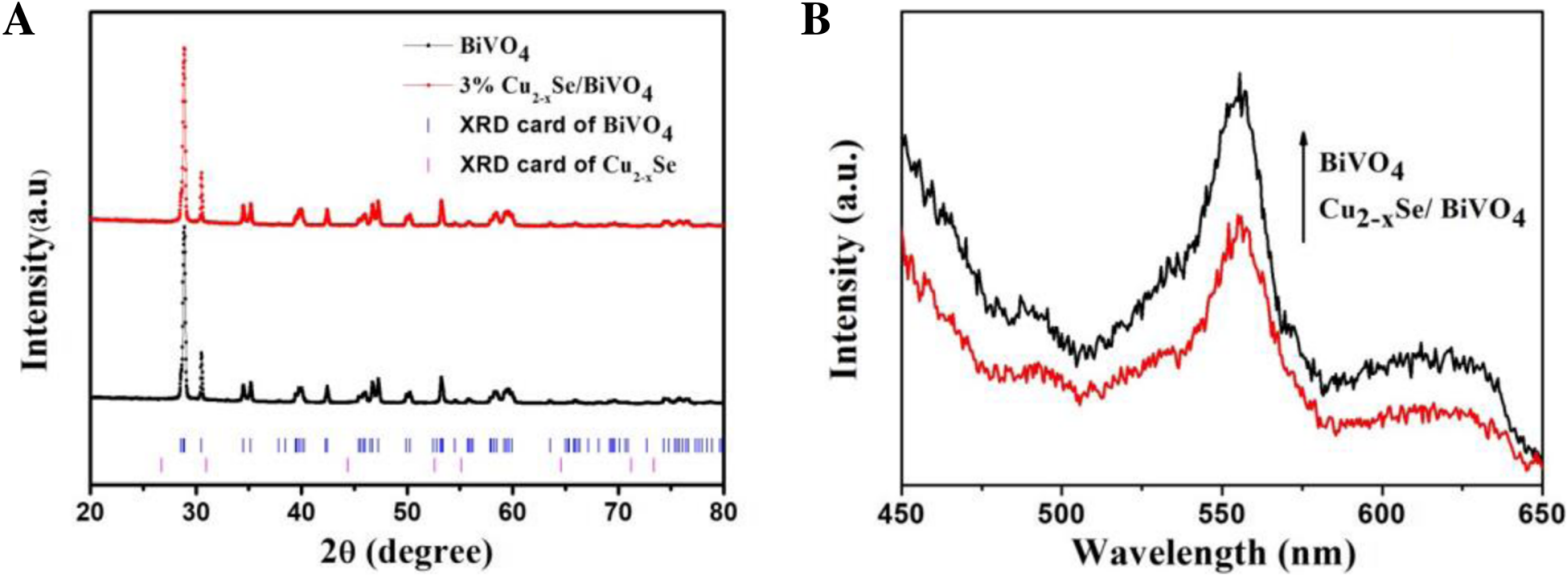
The XRD data for BiVO_4_ and 3% Cu_2−*x*_Se/BiVO_4_ (**a**), the PL spectra for BiVO_4_ and Cu_2−*x*_Se/BiVO_4_ composites (**b**)

Figure 4a shows the XRD data for BiVO_4_ and 3 wt% Cu_2−*x*_Se/BiVO_4_ composite, which exhibits that the BiVO_4_ has a monoclinic crystal structure. It can be seen that the crystal structure of BiVO_4_ does not change when BiVO_4_ is combined with Cu_2−*x*_Se. This may be due to the fact that the content of Cu is relatively too small to be detected by XRD. Photoluminescence measurement is a general way to explore the separation and combination of electrons and holes. The relatively low luminescence intensity means a high electron-hole separation efficiency [27, 28]. Figure 4b shows the PL spectra for BiVO_4_ and Cu_2−*x*_Se/BiVO_4_ composites. After BiVO_4_ is combined with Cu_2−*x*_Se, the relative luminescence intensity of the Cu_2−*x*_Se/BiVO_4_ composite is lower than that of BiVO_4_, which indicates that the Cu_2−*x*_Se/BiVO_4_ composite has higher electron-hole separation efficiency after the combination of BiVO_4_ and Cu_2−*x*_Se.

**Fig. 5:**
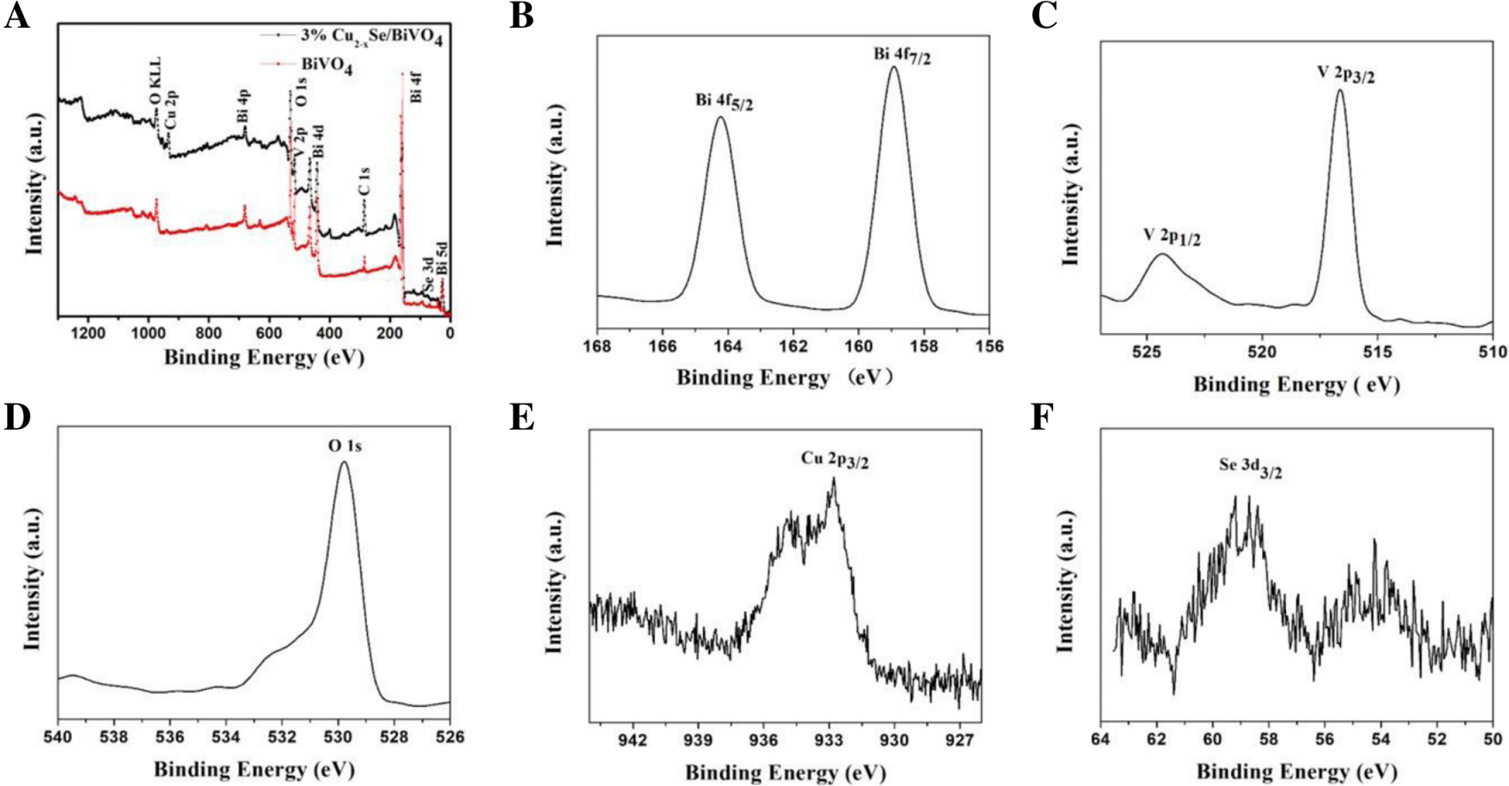
The XPS spectra of Cu_2−*x*_Se/BiVO_4_ composite. **a** Survey, **b** Bi, **c** V, **d** O, **e** Cu, and **f** Se

The surface chemical state plays an important role in determining photocatalytic performance. So XPS is used to analyze the surface element valence of the Cu_2−*x*_Se/BiVO_4_ composite. Figure 5a is the XPS survey spectrum of the Cu_2−*x*_Se/BiVO_4_ composite and pure BiVO_4_, from which characteristic energy of Bi, V, O, Cu, and Se can be observed for Cu_2−*x*_Se/BiVO_4_, and characteristic energy of Bi, V, and O can be observed for BiVO_4_. The peaks of 159.1 and 164.1 eV can be attributed to the binding energies of Bi 4f_7/2_ and Bi 4f_5/2_, respectively (Fig. 5b), which are derived from Bi^3+^ in BiVO_4_ [29]. The peaks of 517.0 eV and 525.0 eV correspond to V 2p_3/2_ and V 2p_1/2_ band respectively (Fig. 5c), which are derived from the V^5+^ of BiVO_4_. The peak of 530.2 eV can be attributed to O 1 s in BiVO_4_ (Fig. 5d) [30, 31]. The two peaks of 58.6 eV and 53.8 eV correspond to Se 3d_3/2_ and Se 3d_5/2_, respectively (Fig. 5e) [32]. The Cu 2p_3/2_ peak located at 931.9 eV corresponds to Cu^0^ or Cu^I^ (Fig. 5f) [33].

**Fig. 6:**
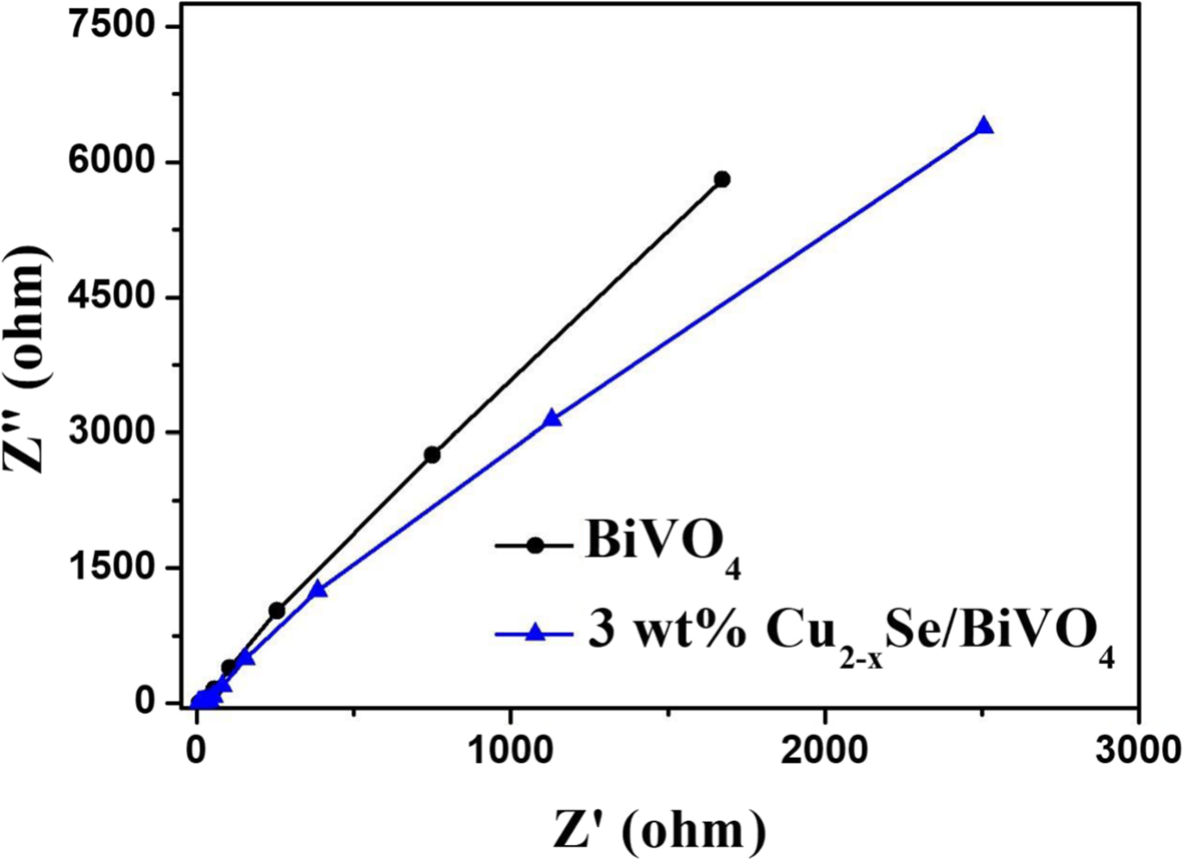
The EIS for BiVO_4_ and Cu_2−*x*_Se/BiVO_4_ under visible light irradiation in 0.5 M Na_2_SO_4_ solution

To further illustrate the separation efficiency of photo-generated electrons and holes, the sample was subjected to EIS analysis. As shown in Fig. 6, the EIS Nyquist diagram of Cu_2−*x*_Se/BiVO_4_ has a smaller arc radius than Cu_2−*x*_Se, indicating that Cu_2−*x*_Se/BiVO_4_ composites have smaller charge transfer resistance and faster interface electron transfer. [34, 35]

**Fig. 7:**
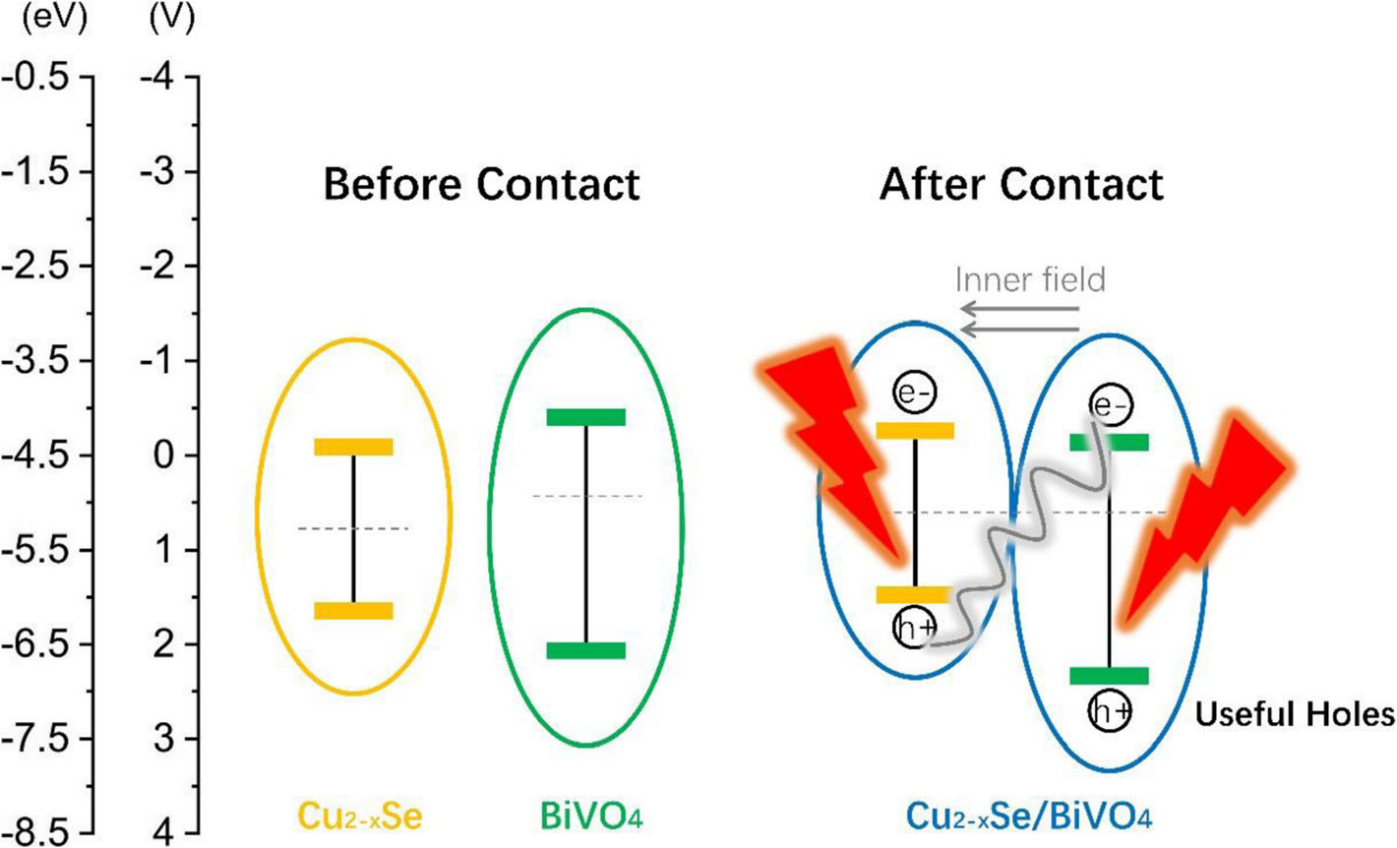
The schematic diagram of photocatalytic mechanism

The reason why Cu_2−*x*_Se/BiVO_4_ composite exhibits high efficiency is explained as follows. As illustrated in Fig. 7, the Fermi level of Cu_2−*x*_Se and BiVO_4_ disagrees with each other. As a result, after the BiVO_4_ semiconductor surface is compounded with CuSe, the charges will be redistributed. Cu_2−*x*_Se has larger work function and lower Fermi energy, so electrons flow to Cu_2−*x*_Se from BiVO_4_ while holes flow the other way around. As a result, the Cu_2−*x*_Se is negatively charged and BiVO_4_ is positively charged until the Fermi level is equal. Meanwhile, the band structure of both materials will bend corresponding to the movement of Fermi levels. Another effect of the redistribution of carriers is the building of an inner field pointing from BiVO_4_ to Cu_2−*x*_Se. Both the Fermi level movement and inner field form the so-called S-scheme heterojunction between Cu_2−*x*_Se and BiVO_4_ [36]. Under illumination, electrons and holes are excited in both materials. In this type of heterojunction, however, the photo-generated electrons in BiVO_4_ and photo-generated holes in Cu_2−*x*_Se will recombine preferentially, due to the band bending and inner field, leaving useful holes in BiVO_4_. The useful holes possess higher energy level, which can benefit the generation of •OH species. These •OH species can break down long chains of organic matter into small molecules. The above results indicate that loading Cu_2−*x*_Se on the surface of BiVO_4_ can enhance the visible light photocatalytic activity.

## Conclusion

In summary, the Cu_2−*x*_Se/BiVO_4_ composites have been successfully prepared and examined for degrading organic pollutions. Experimental data shows that the photocatalytic activity is largely improved after the combination. The photocatalytic efficiency of 3 wt% Cu_2−*x*_Se/BiVO_4_ composite is 15.8 times that of pure BiVO_4_. Furthermore, after adding low concentration H_2_O_2_, RhB can be completely degraded within 50 min. The SEM and XPS results confirm the presence of Cu_2−*x*_Se in the Cu_2−*x*_Se/BiVO_4_ composites. The results of photoluminescence indicate that the Cu_2−*x*_Se/BiVO_4_ composites have higher electron-hole separation efficiency. The results of EIS indicate that Cu_2−*x*_Se/BiVO_4_ composites have smaller charge transfer resistance and faster interface electron transfer. This work shows that Cu_2−*x*_Se is an effective co-catalysis for the development of new composite semiconductor photocatalysts.

## Abbreviations

RhB: Rhodamine B
SEM: Scanning electron microscope
XRD: X-ray diffraction
